# Exploring of spectroscopic, dielectric, and bioactivity performance of bioglass/sodium alginate-PVP loaded-Amoxicillin/Clavulanic Acid microspheres for bone tissue engineering

**DOI:** 10.1038/s41598-025-96590-7

**Published:** 2025-05-02

**Authors:** Ahmed M. Bakr, Amany M. El Nahrawy, A. M. Mansour, Ali B. Abou Hammad

**Affiliations:** 1 Spectroscopy Department, Physics Research Institute, National Research Centre, 33 El-Bohouth St., Dokki, 12622 Giza Egypt; 2 Solid State Physics Department, Physics Research Institute, National Research Centre, 33 El Bohouth St., Dokki, 12622 Giza Egypt

**Keywords:** Bioglass, Sodium alginate, PVP, Amoxicillin/Clavulanic Acid, Bioactivity, SBF, Biophysics, Physics

## Abstract

This study aims to develop an innovative drug delivery bio-system using bioglass (BIOGLASS) and biopolymers of Sodium Alginate (SA) and polyvinylpyrrolidone (PVP) in microsphere form as a carrier for Amoxicillin/Clavulanic Acid drug. In this work BIOGLASS/SA-PVP and Amoxicillin/Clavulanic Acid loaded BIOGLASS/SA-PVP microspheres (0%, 5%, 10%, and 15%) were synthesized using the ion crosslinking method technique. The fabricated microspheres were analyzed using FT-IR, FESEM/EDX, and XRD confirming the *in-vitro* examination. XRD and FTIR data demonstrate the effective creation of the apatite layer and the appearance of new apatite peaks at both 605 cm^−1^ and 565 cm^−1^, distinguishing the prolonged vibrations associated with the ${\text{PO}}_{4}^{-3}$ group. SEM images reveal that the prepared bio-beads have a spherical shape, with sizes falling in the micro-scale. The dielectric constant (εʹ), the dielectric loss (εʺ), and the AC conductivity (σ) were slow at the frequency range of 4 Hz to 8 MHz at room temperature. The antibacterial examinations of the fabricated microspheres were performed employing agar diffusion procedure against the clinical pathogens Gram^+^ and Gram^-^ bacteria. The SBF (simulated body fluid) experiments display the formation of a hydroxy appetite coating on the microsphere’s surfaces that approves their significant bioactivity. Furthermore, antimicrobial results of BIOGLASS/SA-PVP/Amoxicillin/Clavulanic Acid microspheres reveal a notable impact on the antimicrobial performance. The *in-vitro* tests established that fabricated bio-microspheres are a promising opportunity for bone tissue engineering (substitutes and regeneration), signifying their promise for bone application.

## Introduction

In recent decades, considerable focus has been directed towards the application of synthetic inorganic amorphous biomaterials, specifically bioglasses ^1,2^, due to their broad to their broad applications across diverse technological domains, including drug delivery, osteoconductive uses, biosensors, and scaffolds for tissue engineering ^3^. In the realm of the quest for innovative biomaterials that can seamlessly integrate with the biological environment and enhance regenerative processes remains a focal point of research ^4–6^. Since the researchers have extensively employed bioglass materials in clinics, sparking increased interest in developing regeneration scaffolds which support the load with the bone and stimulate regenerative mechanisms. The effective implant structures must possess bioactivity and toughness to imitate cancellous bone with open porosity ^7–9^.Despite the widespread use of ceramics, their brittleness, fragility, and limited crack resistance prompted the search for alternatives due to their non-bioactive nature. Hence, the synthesis of bio-systems, which are chemically and crystallographically similar to bone materials, is crucial for developing biomaterials that can efficiently integrate with natural bone systems^10,11^. Bioglass is a bioactive glass that has gained significant attention and application in various fields, particularly biomedical engineering and regenerative medicine ^12,13^. Dr. Larry Hench pioneered the production of this feature material during the 1960s. Its composition is based on the presence of (CaO), (Na_2_O), (P_2_O_5_), and (SiO_2_) ^9,14,15^. The distinctive feature of Bioglass lies in its ability to bond with living tissues, promoting integration and regeneration.

Bioactive glasses (BIOGLASSs) have emerged as promising materials for tissue regeneration due to their excellent bioactivity, biocompatibility, and antibacterial properties against common pathogens such as Escherichia coli and Staphylococcus aureus. The sol–gel synthesis of bioglasses offers advantages over conventional melting-quenching techniques by producing materials with higher porosity and specific surface area, which enhance their bioactivity and osteoconductivity. Recent studies highlight the role of different BIOGLASS compositions, including strontium-doped and silica-based variants, in improving mechanical strength and biological responses. Moreover, the incorporation of poly(L-lactide-co-ε-caprolactone) (PLCL) as a compatibilizer has been shown to reduce heterogeneity in nanocomposites, leading to improved structural and functional properties. Additionally, mesoporous bioactive glasses (MBIOGLASSs) are gaining attention in biomedical applications, particularly for their potential role in cancer diagnostics and targeted therapies, including photothermal treatments. Despite their promising attributes, the clinical application of MBIOGLASSs remains underexplored, necessitating further research into their therapeutic mechanisms. Overall, the ability of bioactive glasses to promote cell proliferation and differentiation underscores their significance in hard tissue engineering, making them strong candidates for bone reconstruction and implant development ^16–19^.

El Nahrawy et al. ^9^ found that in SiO_2_–P_2_O_5_ binary oxides, the variation of P_2_O_5_ structural properties is caused by chemical interactions through the sol–gel procedure, including the generating of Si–O–P bonds or Si–O– groups at the SiO_2_–P_2_O_5_ interface ^9,20^. The growth of both P_2_O_5_ and CaO particles in the SiO_2_ matrix is of interest for spectroscopic and bone engineering purposes.

Sodium alginate is a natural compound produced from seaweed and employed in the dental, pharmaceutical, and food industries ^21,22^. It has thickening, gelling, and effective film-forming properties, making it important for creating various products and refining different processes ^23,24^. Sodium Alginate (SA), a biopolymer obtained from brown seaweed, is a natural carbohydrate consisting of mannuronic (M) and guluronic (G) acid units. It provides a biocompatible and biodegradable matrix, making it ideal for varied biomedical applications, containing drug delivery and tissue engineering ^22,23^. (PVP) is a synthetic polymer from the class of polyvinyl polymers ^25,26^. It is a multipurpose and widely used composite in various industries applications, including industrial processes, pharmaceuticals, packing, and cosmetics to food. PVP chemical structure consists of retelling units of vinylpyrrolidone, making it a highly stable and water-soluble polymer ^27,28^. Surface coatings are among the effective procedures for modifying the bioactivity and biodegradation of organic inorganic-based nanocomposites planned for drug delivery and temporary transplant bio-applications ^4,29,30^. Spectroscopic analysis provides invaluable insights into the biomaterials structural and chemical composition, allowing for a comprehensive understanding of their behavior at the molecular level ^31,32^.

Bioglass, known for its bioactive properties, and SA, a biocompatible polymer, synergistically contribute to the formulation of microspheres with tailored drug release profiles and improved bioavailability ^33,34^. This study explores the bioactivity of these microspheres, assessing their ability to not only deliver Amoxicillin/Clavulanic Acid efficiently but also to interact with biological systems, thus opening avenues for advanced drug delivery systems with enhanced therapeutic outcomes ^23,35^. In our bio-microspheres, Sodium Alginate forms a hydrogel that encapsulates the Bioglass and PVP, ensuring a controlled release of incorporated Amoxicillin/Clavulanic Acid. Additionally, the gelation of sodium alginate with calcium ions enhances the structural properties and stability of the bio-microspheres. Also, Polyvinylpyrrolidone (PVP) is a synthetic polymeric material with a repeating N-vinylpyrrolidone monomer. It acts as a binder and stabilizer and enhances the solubility and dispersion of Bioglass particles within the alginate matrix in the bio-microsphere formulation ^25,36^.

Bioglasses are typically synthesized using the traditional melting method, which is considered simple and suitable for large-scale production ^37–39^. However, this approach is constrained by the evaporation of the volatile component P₂O₅ at high temperatures ^40^. In contrast, the sol–gel technique has emerged as a widely studied alternative for bioglass fabrication in recent years. Templated silica-based bioglass has diverse advantages that variety it a fascinating research area ^40^.In previous studies on 45S5 bioglasses, sodium and calcium nitrates with nitric acid, have been commonly used in sol–gel processes for bioactive glass ^40–42^. These materials are favored due to their cost-effectiveness, excellent solubility, and ease of thermal decomposition. However, heat treatments over 600 °C are required to eliminate nitrate byproducts, which can pose potential risks to living cells^40–42^.

In this study, we use a CaO–P_2_O5–SiO_2_ template for the sol–gel process. This approach avoids the use of higher nitrate compositions, allowing for better control over the microstructure and properties of the bioglasses with limited potential risks to living cells.

This unique combination of an organic matrix and Bioglass could enhance the properties of the CaO–P_2_O_5_–SiO_2_ matrix, offering new possibilities for structural and dielectric properties, as well as the development of advanced biomaterials ^43,44^. The incorporation of P_2_O_5_ and CaO particles is particularly noteworthy as it may improve the bioactivity and sensitivity of the microsphere composites, making it highly relevant for bone regeneration and repair ^34,45,46^. Typically, Bioglass is composed of SiO_2_, CaO, and P_2_O_5_^7,34,47^. When Bioglass is immersed in body fluids, it undergoes a set of surface responses that lead to the construction of a hydroxyapatite coating, similar to the mineral phase of bone.

Additionally, the spectroscopic properties of the composite could lead to innovative applications in bio-imaging and diagnostics, further underlining the importance of this research.

This study delves into the promising frontier of biomaterial science by exploring the spectroscopic characteristics and bioactivity performance of Amoxicillin/Clavulanic Acid-loaded bioglass/SA-PVP microspheres. These intricate structures, designed with precision, hold the potential to revolutionize bone tissue engineering through a multifaceted approach. The formation of microspheres-based drug delivery systems allows control through specific target sites and the drug release line by accurately supporting the combination of various nanoparticles with polymer-drug systems. This exploration aims to unravel the spectroscopic signatures embedded within the Amoxicillin/Clavulanic Acid-loaded bioglass/ SA-PVP microspheres, shedding light on their structural intricacies and paving the way for optimized performance in the complex milieu of bone tissue ^21,48^. Furthermore, the study investigates the performance of these microspheres, and their ability to stimulate favorable responses from the surrounding biological environment. Amoxicillin/Clavulanic Acid, a well-known antibiotic, adds a layer of functionality by potentially imparting antimicrobial properties to the biomaterial. The combination of pharmaceutical agents with the Bioglass/SA-PVP matrix not only supports regenerative processes but also reduces infection risks, which is crucial in bone tissue engineering ^45,49^.

Unlike conventional bioglass-based drug delivery systems, this research introduces a dual-function microsphere composite that integrates bioactivity and antimicrobial efficacy. The use of a sodium alginate-PVP matrix optimizes drug encapsulation and sustained release, while the modified bioglass composition enhances osteointegration. Additionally, the study uniquely correlates dielectric properties with antimicrobial performance, providing insights that could inform future bioactive material designs.

This study aims to thoroughly investigate the microstructural, spectroscopic characteristics, dielectric properties, and bioactivity of Amoxicillin/Clavulanic Acid-loaded Bioglass/Sodium Alginate-PVP microspheres for bone tissue engineering. These microspheres are designed to promote tissue growth and minimize infection risks, to optimize bone repair. The dielectric properties are important for tissue integration, cell behavior, and overall biocompatibility. By combining the bioactive properties of bioglass with the antimicrobial benefits of Amoxicillin/Clavulanic Acid, these microspheres offer controlled drug delivery and enhanced tissue growth. This synergistic approach aspires to provide more effective solutions for bone tissue engineering, reducing infection risk and optimizing bone repair. This research paves the way for innovative bio-material solutions in the field of bone regeneration. The ultimate goal is to create advanced Amoxicillin/Clavulanic Acid-loaded bioglass/SA-PVP microspheres for bone regeneration, providing more effective solutions for bone tissue engineering and reducing infection risk.

## Experimental work

### Materials and methods

Sodium Alginate (Panreac Química SLU), Polyvinylpyrrolidone (Mw = 360,000) **(**Sigma-Aldrich Co USA), and calcium chloride powder CaCl_2_(Sigma-Aldrich) were employed in the microsphere fabrication. Tri-Ethyl-Phosphate (C_6_H_15_O_4_P, Mw = 182.15 g/mol, Sigma-Aldrich), Calcium Nitrate Tetrahydrate (Ca(NO_3_)_2_ 0.4H_2_O, Mw = 236.149 g/mol, Sigma-Aldrich), Ethanol (Sigma-Aldrich), Tetra-Ethyl-Ortho-Silicate (TEOS: C8 H_20_O_4_Si, Mw = 208.33 g/mol, Sigma-Aldrich), and nitric acid (HNO_3_) were bought from Merck Inc (Darmstadt, Germany), and Amoxicillin/Clavulanic Acid. All other chemicals required in the synthesis of simulated body fluid (SBF) and phosphate-buffered saline (PBS) were brought from Sigma-Aldrich.

### Formation of bioactive glass

The bioactive glass design was produced through the sol–gel method, following the composition ratios of CaO (32 wt%), SiO₂ (57 wt%), and P₂O₅ (11 wt%). Distinct amounts of 43.5 ml of TEOS (tetraethyl orthosilicate) and triethyl phosphate (6 ml), dissolved in ethanol, were mixed with nitric acid (6 ml–22 M). At room temperature, the mixture of TEOS/triethyl phosphate/ethanol was pre-hydrolyzed for 1 h in ethanol–water solution, nitric acid as catalysis ^4,50–52^. So, the P_2_O_5_–SiO_2_ sol was obtained. Next, 3.626 g of Ca(NO_3_)_2_ was disbanded in H_2_O (20 ml) and incorporated into the TEOS/triethyl phosphate solution. The mixture was stirred vigorously for 1.5 h to ensure complete hydrolysis. Then, the acidifying P_2_O_5_–SiO_2_–CaO sol was kept for 10 h to induce the gelation of P_2_O_5_–SiO_2_–CaO sol ^53^. The obtained powders were desiccated for 48 h at 150 °C and heated at 500 °C to eliminate any remaining water and nitrate compounds. The powders were then ground using an agate mortar to break up agglomerates before being incorporated into the SA/PVP matrix.

### Formation of drug-loaded BIOGLASS/SA-PVP microspheres nanocomposite

**Fig. 1:**
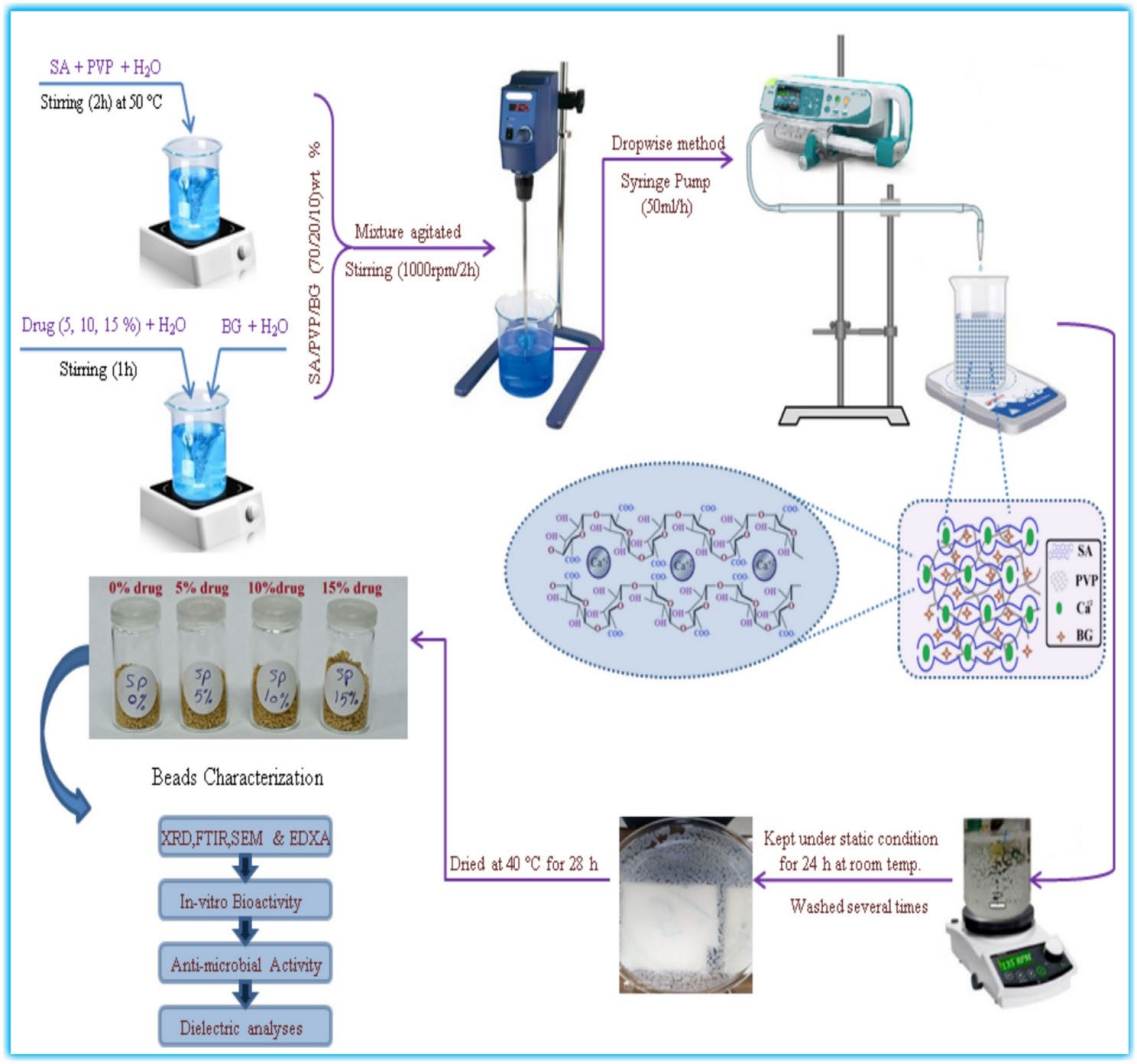
Illustration displaying the fabrication stages of drug-loaded microspheres.

Microspheres containing varying drug concentrations were produced using a constant weight ratio of SA, PVP, and BIOGLASS (7: 2: 1). The dissolved biopolymers were mixed together under gentle mechanical stirring at 40 °C for 90 min, subsequently the BIOGLASS was slowly added following ultrasonic dispersion. Different ratios of Amoxicillin/Clavulanic Acid (0%, 5%, 10%, and 15%) were loaded into the Bioglass/SA-PVP mixture and left for 75 min (Fig. 1 and Table 1). The obtained mixtures were mechanically stirred for 3 h using an overhead High Torque-Stirrer homogenizer. Following that, the acquired mixture of BIOGLASS/SA-PVP filled with the drug was gradually dripped employing a syringe pump into CaCl_2_ solution (0.5 M, 500 ml) with a rate of (50 ml/h) to yield the hydro-gel microspheres and moved at 120 rpm on a stirring at 30 °C for 2 h. To finalize the formation and solidification of the gel microspheres, the prepared microspheres were immersed in the crosslinking solution and left undisturbed at room temperature for one day. Afterward, the fabricated microspheres were taken out of the solution, absolutely rinsed with double-distilled water, and left to air-dry at 42 °C for 48 h. Ultimately, the dried samples were collected and stored in sealed vials.

**Table 1:** Chemical composition of Drug-loaded BG/SA-PVP microspheres nanocomposite.

Sample Code	Amoxicillin/Clavulanic Acid wt% g (%)	SA	PVP	BG	SA/PVP/BG wt%	
SP-0	0	0	1.4 g	0.4 g	0.2 g	7: 2: 1
SP-5	5	0.105 g	1.4 g	0.4 g	0.2 g	7: 2: 1
SP-10	10	0.222 g	1.4 g	0.4 g	0.2 g	7: 2: 1
SP-15	15	0.353 g	1.4 g	0.4 g	0.2 g	7: 2: 1

### Characterization

The X-ray diffraction (XRD) analysis of the BIOGLASS/SA-PVP microspheres containing the Amoxicillin/Clavulanic Acid was performed using a Panalytical Empyrean device. Utilizing CuKα radiation with a wavelength of 1.54 Å, the scan was conducted over a 2θ range of 5 to 60°. The FTIR examination of the prepared microspheres nanocomposites was carried out using a Bruker Vertex 70 V spectrometer. This technique supports determining the functional groups in the bio-microspheres through the attenuated total reflectance (ATR) mode. Confirm that the microspheres are completely dry before placing them directly on the ATR crystal, and the spectra were recorded within the range of 4000 to 400 cm^−1^.

Besides, scanning electron microscopy (Quanta-FEG 250-Japan), without samples gold coating, was used to visualize the fabricated microspheres nanocomposites incorporating Amoxicillin/Clavulanic Acid. Attach the dried bio-microsphere samples to SEM sample holders using adhesive carbon tape. Apply a thin coating (gold) to the samples. A scanning electron microscope (SEM) equipped with Energy-Dispersive X-ray (EDX) analysis was employed to determine the elemental composition of the prepared nanocomposites. Obtain the EDX spectrum of the fabricated microspheres to analyze and confirm the element’s presence and distribution, like carbon (C), silicon (Si), calcium (Ca), phosphorus (P), oxygen (O), and other elements relevant to the acquired material.

The dielectric analysis was Performed by Hioki LCR IM3536. The microsphere samples were crushed and pressed into a disk with a radius of 6.5 mm and thickness. The dielectric constant (εʹ), dielectric loss (εʺ), and the AC conductivity (σ) were obtained from:

1 $${\varepsilon }{\prime}= C\frac{t}{A {\varepsilon }_{0}},$$

2 $${\varepsilon }^{"}=\text{tan}\left(\delta \right)*{\varepsilon }{\prime},$$

3 $${\sigma }_{ac}= G\frac{t}{A},$$

A is the electrode radius, t is the sample thickness, C is the experimental capacitance, ε_0_ is the free space permittivity, tan (δ) is the dissipation factor (practically obtained from device results), and G is the experimental conductance ^54^.

### Antimicrobial efficacy

The standard agar well diffusion procedure was employed to evaluate the antimicrobial significance of the fabricated bio-microspheres. Following the procedures outlined by Perez et al., a variety of pathogenic bacteria were selected as test organisms ^55–57^. To obtain purified cultures, the chosen strains were subcultured in nutrient broth and then inoculated onto Petri plates containing Muller-Hinton agar. In order to evaluate the antibacterial effects, 100 μl of each tested sample was added to circular wells (7 mm in diameter) created on the plates using a sterile cork borer. After that, the established plates were incubated overnight at 37 °C, and the resulting inhibition zones were noted afterward. To investigate the antibacterial performance, we used the agar well diffusion technique and employed Gram^-^ strains (*Escherichia coli*, and *Klebsiella pneumoniae*) as well as Gram^+^ bacterial strains (*Enterococcus faecalis*, *Staphylococcus haemolyticus*, and *Staphylococcus aureus*). On the other hand, the fungal strains *Candida albicans* and *Candida auris* were grown on an appropriate agar plate in a liquid medium. The mycelial fragments were harvested, purified, and resuspended to produce the fungal inoculum. In order to cultivate the yeast species, Sabouraud Dextrose Agar (SDA) was employed, and the plates were then incubated at 28 °C, the optimal temperature for fungal growth, for 48–72 h. The experiment was repeated three times, and the appearance of a clear inhibition zone close to the sample in the inoculated Petri dishes signified the antimicrobial activity of the fabricated bio-microspheres.

### In vitro* bioactivity study*

To evaluate the bio-microspheres capability to constitute hydroxyapatite on their surface when immersed in a solution that mimics human blood plasma. Following the standard procedure outlined by Kokubo and Takadama, the bioactivity evaluation of the organized BIOGLASS/SA-PVP and Amoxicillin/Clavulanic acid-loaded microspheres was assessed in vitro by immersing the fabricated samples in simulated body fluid (SBF) ^58^. Weigh a known number of bio-microspheres. The prepared samples, at a concentration of 2.5 g/L, were submerged in SBF solution in sealed containers at 37 °C for 28 days. Upon release from SBF, rinse the microspheres lightly with deionized water to remove any loosely bound surface deposits. Dry the samples at room temperature or in a desiccator. Using XRD and FTIR to determine crystalline phases and chemical bonds for the bioglass/SA-PVP loaded Amoxicillin/Clavulanic Acid bio-microspheres’ surface to confirm hydroxyapatite formation and EDX to examine the elemental composition and confirm the presence of calcium and phosphorus, indicative of hydroxyapatite formation. The SBF immersion process, providing valuable insights into their bioactivity performance, resulted from bioglass-PVP-Sodium Alginate bio-microspheres.

## Results and discussion

### XRD investigation

**Fig. 2:**
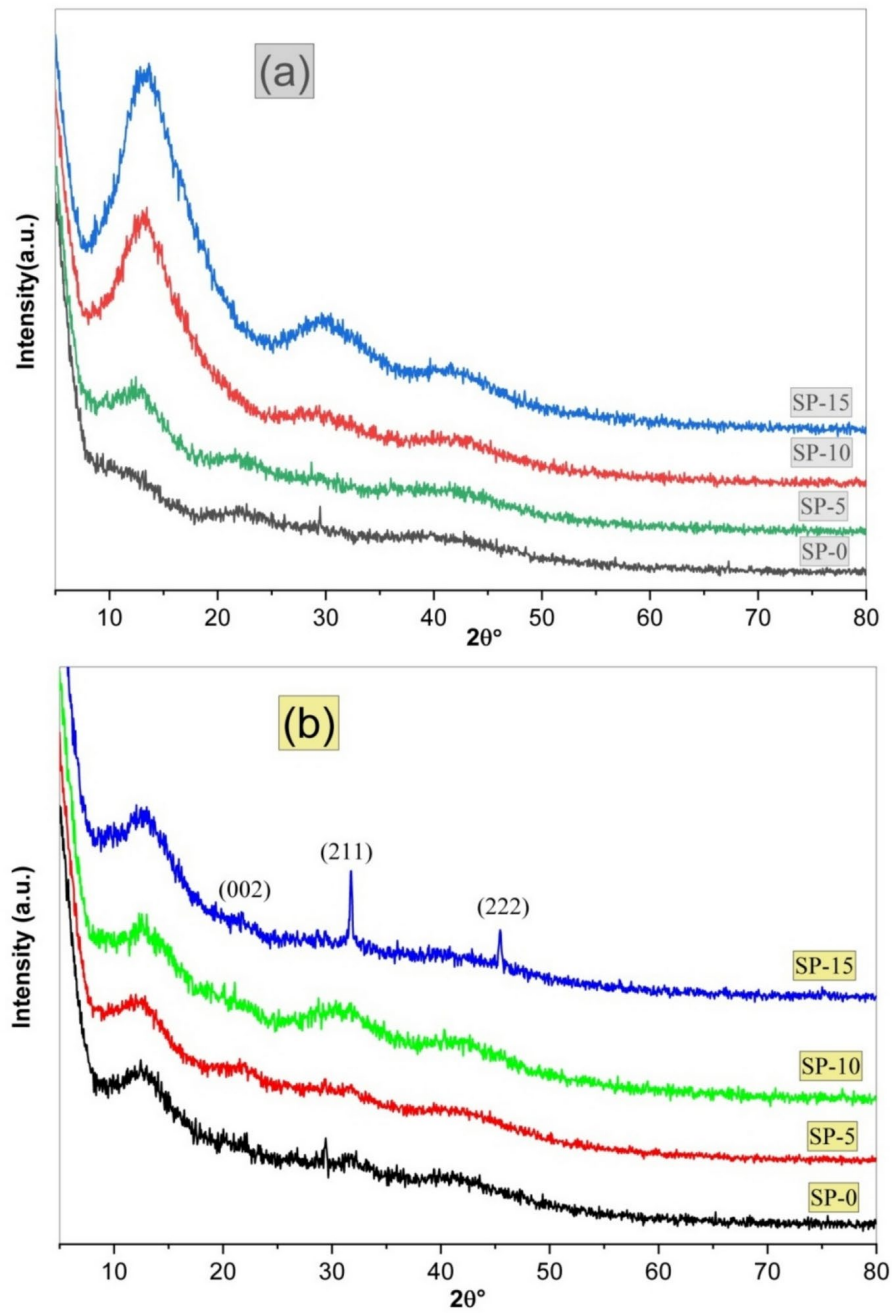
XRD patterns of the fabricated microspheres SP-0, SP-5, SP-10, & SP-15 nanocomposites (**a**) before and (**b**) after immersion in SBF.

The crystallographic alterations, degree of crystallinity, and phase purity of the prepared microsphere samples were assessed employing the XRD methodology. Figure 2 displays the XRD outlines of the prepared microspheres nanocomposites (a) earlier and Fig. 2b for the microspheres submerged in SBF solution for 28 days. Before immersion in SBF and as shown in Fig. 2a, the patterns don’t present any identified diffraction profile excluding wide peaks found in the range 2θ (10–40°). These broad peaks are mainly associated with the amorphous character of both bioglass and biopolymers, while the drug concentration rises gradually, the level of crystallinity incrementally increases ^37,59^. The fundamental idea implies the biological bustle of the resulting bio-microspheres for attaching to live bone is the construction of the hydroxyapatite coating on the microsphere’s surface. The in vitro study, using SBF solution, was achieved to assess the microsphere’s bioactivity. The prepared microspheres were kept for 28 days at 37 °C while immersed in SBF, and the precipitating apatite was recognized by XRD investigation. This result indicates that loading varying amounts of Amoxicillin/Clavulanic Acid into the investigated bioglass/SA-PVP microsphere system supports its internal structure (Fig. 2a). The XRD patterns of the composites containing Amoxicillin/Clavulanic Acid reveal their composite nature, characterized by an amorphous phase. This finding aligns with previous research by Pon-On et al.^60^, which demonstrated that the modified CaO–SiO_2_–P_2_O_4_ has a low-intensity crystalline structure typical of nano-bioactive glass ^39,59^. Our results align well with the findings reported in the literature ^38,39,42,59,61^.

 Figure 2b illustrates the XRD profile of the fabricated microspheres, after 28 days of submersion in SBF. Notably, the spectra reveal the exact manners found in the microsphere’s structure before submersion in the SBF, except for the appearance of two identified bands observed at 2θ = 31.7 and 45.5° and noticed distinctly in SP-15 samples. Generally, these peaks are owing to apatite structure, relying on the JCPDS (76–0694).

It is promising to observe that the apatite phase was formed at such a lower temperature, producing relative crystalline changes from the amorphous behavior of bioglass/SA-PVP to semicrystalline apatite phase with no obvious CaO and P_2_O_5_ phases can be found. Nevertheless, the virtual intensity of the (211) plane noted in sample SP-15 was more elevated than that observed in all samples, referring to the outstanding crosslinking HAp layer to the microsphere’s surface and the combined effects of higher drug content and bioglass interactions, which means that the drug improves the microsphere’s bioactivity.

The SP-15 sample exhibits a more intense (211) plane in the XRD pattern, indicating a superior hydroxyapatite (HAp) layer due to the combined effects of higher drug content and bioglass interactions. The increased concentration of Amoxicillin/Clavulanic Acid in SP-15 enhances ion exchange and surface reactivity, which promotes apatite nucleation and growth, where the drug molecules can act as nucleation sites and facilitate the formation of hydroxyapatite when immersed in simulated body fluid (SBF). Also, the presence of functional groups such as –COO⁻ and –OH in the drug may interact with Ca^2^⁺ and ${\text{PO}}_{4}^{3-}$ ions, supporting the formation of calcium-phosphate bonds. Therefore, the superior hydroxyapatite layer in SP-15 is due to the synergistic effect of drug incorporation and bioglass dissolution, leading to enhanced ion exchange, nucleation, and crystallization in SBF ^4,8,50,51,62,63^.

The results from the XRD conducted in this work stand well with the recognized results of Bouyer et al. (2000) ^64^. Where Bouyer et al. reported that hydroxyapatite crystallization progresses through an initial amorphous calcium phosphate (ACP) phase, which gradually transforms into a crystalline apatite structure.

### Microspheres morphology

**Fig. 3:**
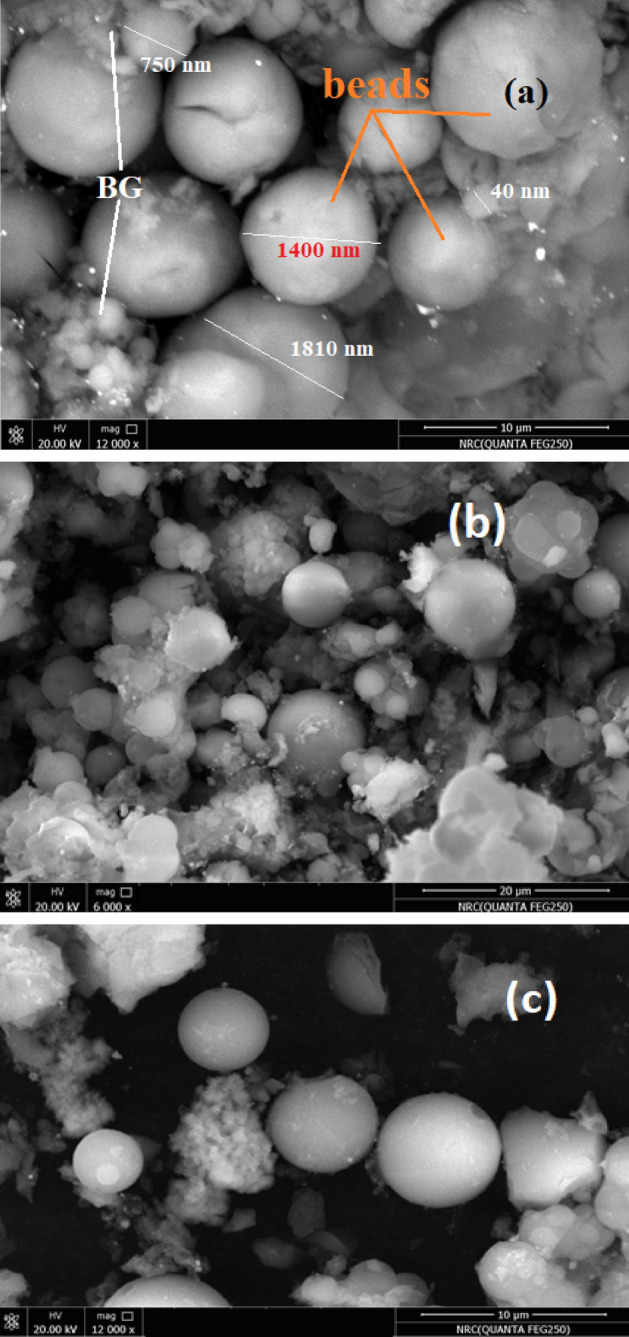

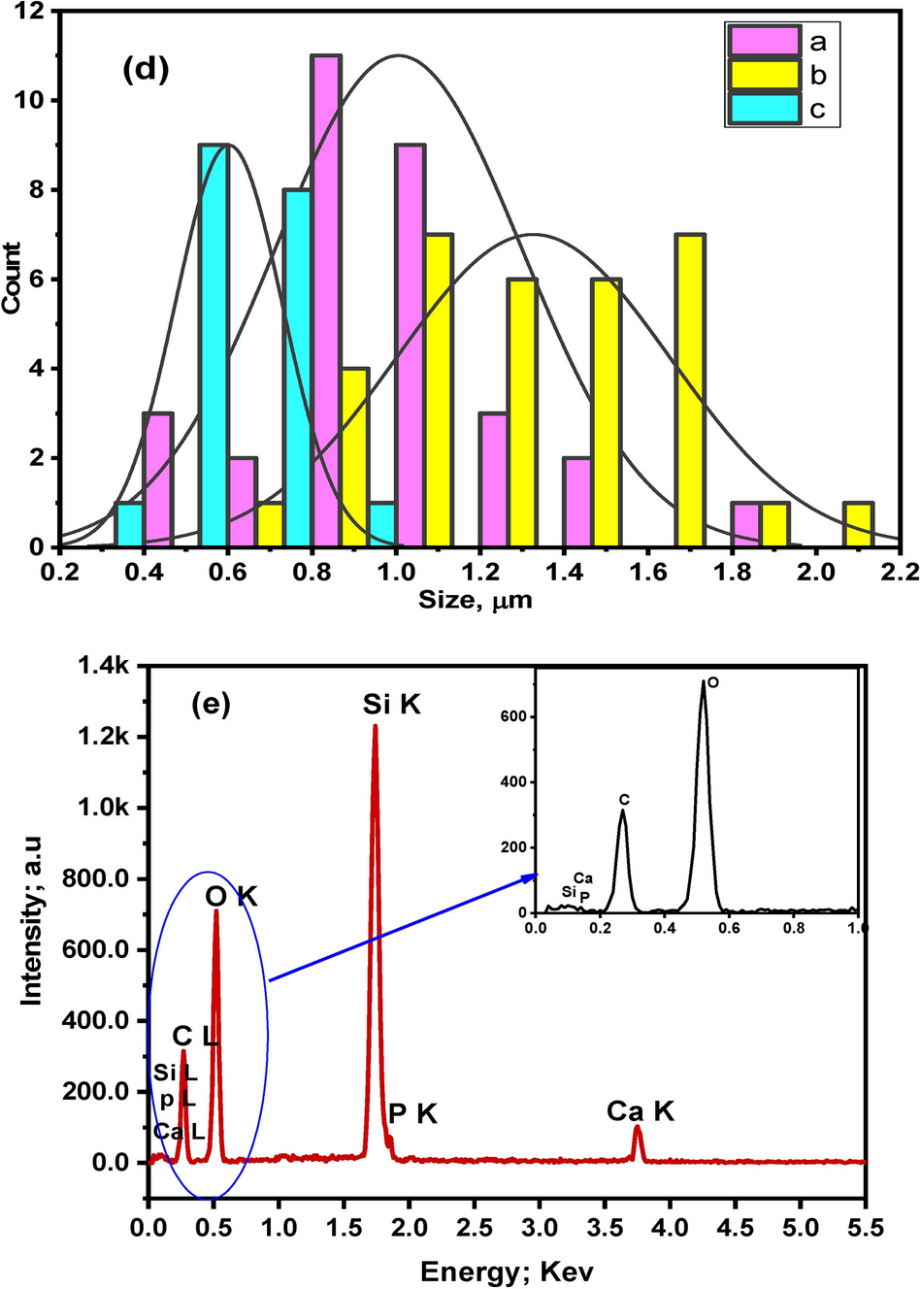
SEM image of the prepared microspheres (**a**) SP-0 and (**b**, **c**) SP-15, (**d**) microspheres size distribution, and (**e**) The EDX spectrum for SP-15.

 Figure 3 demonstrates the SEM micrographs of bioglass/SA-PVP incorporated with 15% of drug, as the images illustrate the surface form of the prepared microspheres. The grain morphology is spherical and well-arranged in the micrograph. The SEM images (Fig. 3b,c) for bioglass/SA-PVP-15% Amoxicillin/Clavulanic Acid bio-microspheres reveal a well-structured and higher stability nanocomposite material, highlighting their potential effectiveness in bone engineering applications. All the samples appear well-matched distribution with an average diameter of the spherical microspheres shape and sizes in the range from 400 to 1800 nm for bioglass/SA-PVA and from 350 to 2000 nm for the sample contained 15% Amoxicillin/Clavulanic Acid (Fig. 3d). The EDX (energy-dispersive X-ray) spectrum of bioglass/SA-PVP-15% Amoxicillin/Clavulanic Acid (Fig. 3e), it can be seen that the produced microspheres contain carbon (C), oxygen (O), phosphate (P), silica (Si), and calcium (Ca). Also, the EDX spectrum verified the elemental composition of the fabricated microspheres, and the appearance of a C band approves the presence of a sodium alginate-PVA polymeric medium.

### FTIR analysis

**Fig. 4:**
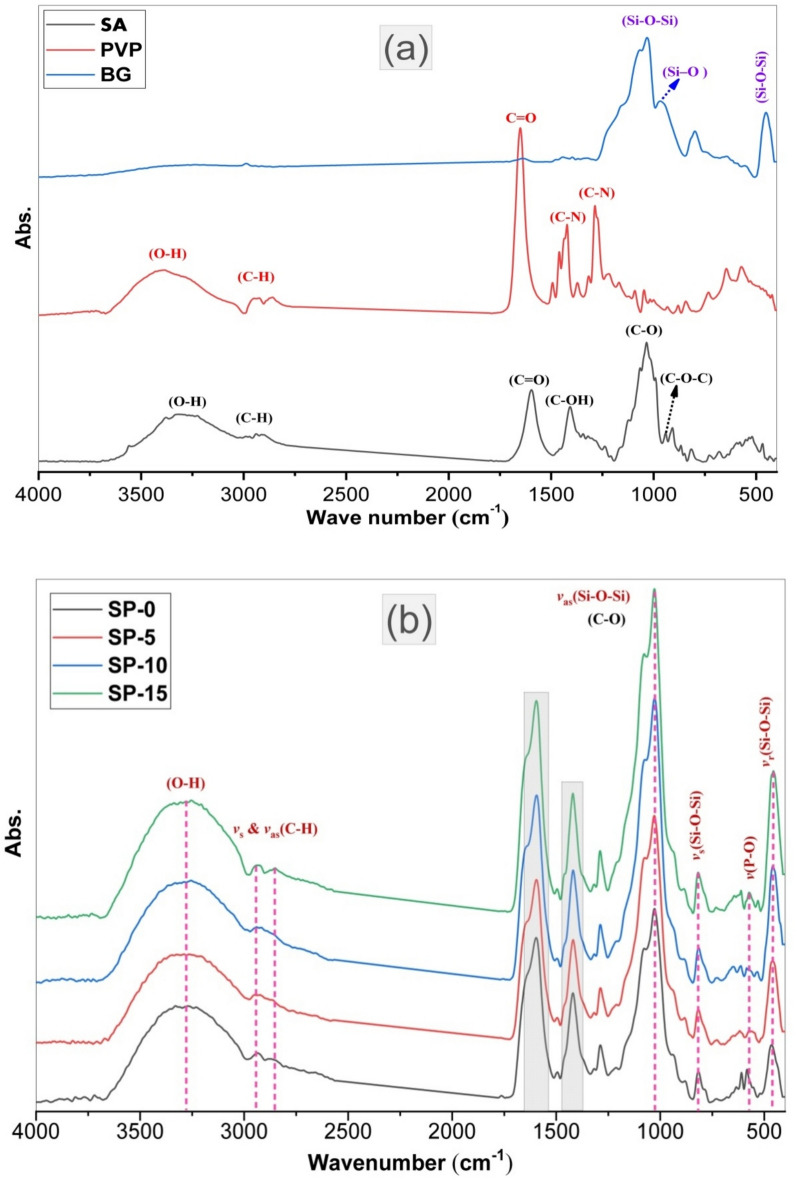

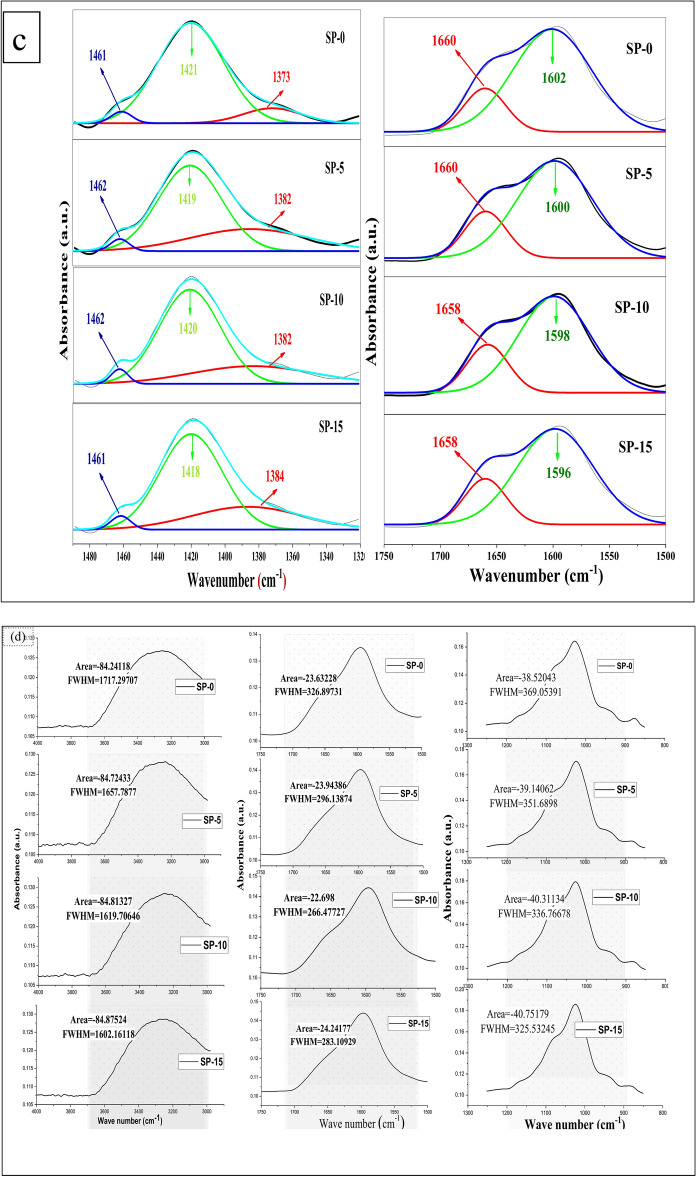
FTIR spectra of (**a**) SA, PVP, and BIOGLASS powders, (**b**) SP-0, SP-5, SP-10, and SP-15 before soaking in SBF (after 28 days), (**c**) deconvoluted spectra at range (1700–1500) and (1480–1320 cm^−1^), (**d**) Area under peaks and FWHM at (3300, 1600 and 1050 cm^−1^).

 Figure 4a FTIR bands of SA, PVP, and bioglass powders, (b) (SP-0), (SP-5), (SP-10), and (SP-15) before submersion in SBF, (c) deconvoluted curve at range (1700–1500) and (1480–1320 cm^−1^), (d) area under peaks and FWHM at (3300, 1600 and 1050 cm^−1^) and, (e) the bio-microspheres after submersion in SBF.

Essentially, FT-IR was employed to recognize the chemical assembly of the natural materials as well as to detect any potential chemical interactions between them. The FTIR profile of SA is described in Fig. 4a. The main characteristic peaks observed at 3000–3550 cm^−1^ aligned to the stretching modes of –OH, while aliphatic (C–H) stretching vibrations appeared at 2950–2840 cm^−1^. The distinct peaks at 1595 cm^−1^ and 1404 cm^−1^ were owing to the asymmetric and symmetric stretching mood of the COO group ^21,22^. The observed vibrations presented at 1038 and 950 cm^−1^ mainly accredited to the widening vibration of (C–O), (C–C–H), and C–O–H deformation ^50,63,65–67^.

 Figure 4a illustrates the principle distinct FTIR peaks of the PVP. The spectrum displays peaks at 3397, 2938, 2862, and 1648 cm^−1^, which are owing to (–OH) stretching, asymmetric of (C–H_2_) of pyrrole ring, symmetric of C–H_2_ of chain, and C=O stretching modes, respectively. The distinct vibrations at 1423 and 1285 cm^-1^ are ascribed to (C–N) bending and C–N wagging modes. The (C–N) vibration presented at 1018 cm^−1^. Also, CH_2_-rock and N–C=O bending modes were displayed at 1049 and 565 cm^−1^, correspondingly ^68–70^. The FTIR spectrum associated to the bioactive glass powder is presented in Fig. 4a. The wide peak placed from 3100 to 3500 cm^−1^is attributed to (–OH) stretching vibration, which is attributed to vigorous hydrogen bonding, both intermolecular and intramolecular in nature. The small peaks positioned from 1639 to 1396 cm^−1^ were related to the (O–H) bending modes of the hydroxyl groups, which are chemically linked to the bioglass ^34^. The fundamental absorption band located around 900–1250 cm^−1^, with maximum intensity at 1033 cm^−1^ is owing to the stretching vibration mode of Si–O–Si. The asymmetric vibration of (Si–O–) is placed at 967 cm^−1^, while the symmetric (Si–O–) stretching is noted at 800 cm^−1^. The medium band observed at 454 is ascribed to the Si–O–Si and Si–O–M (M: Ca, P) bending vibrations ^9,71,72^. The FTIR spectra reveal notable shifts in characteristic functional group vibrations upon crosslinking and drug loading. This shift is consistent across all samples (SP-0, SP-5, SP-10, SP-15), with increasing shifts correlating with higher drug loading. The intensities of the Si–O–Si peaks (1033 cm⁻^1^ and 801 cm⁻^1^) increase with drug loading, suggesting enhanced interaction between bioglass and the polymer matrix ^73,74^. These characteristic vibrations are commonly observed in amorphous silica glasses ^50,51,62,75^.

FTIR spectroscopy examinations were evaluated to perceive the establishment of innovative chemical vibrations or the improvement of present ones, which may be associated with probable interactions between starting materials. Figure 4b displays the FTIR patterns of the prepared samples (SP-0), (SP-5), (SP-10), and (SP-15) before immersion SBF, and generally reveals the major absorption peaks of the ordinary profile of the SA, PVP, and BIOGLASS. Figure 4c demonstrates the deconvoluted curve at range (1700–1500) and (1480–1320 cm^−1^), while Fig. 4d exhibited area under peaks and FWHM at (3300, 1600 and 1050 cm^−1^) respectively. Nevertheless, various distinct peaks are deformed, shifted, or disappeared, which may be attributed to certain chemical interactions between the SA, PVP, BIOGLASS, and drug ^44,47^. The stretching mods band of the O–H group in Na_2_O alginate microspheres seems thinner than SA. This variance occurs due to the crosslinking process in which hydroxyl groups of the alginate participate in CaO to initiate the chelating structure. Additionally, the characteristic peaks at 1595 and 1404 cm^−1^, which are associated to the symmetric and asymmetric stretching modes of the COO group in SA shift to 1602 and 1420 cm^−1^ for calcium alginate microspheres. This manner is attributed to the chemical mechanism between calcium ions (Ca^2+^) of the calcium chloride crosslinker (CaCl_2_) and the carboxyl groups throughout the crosslinking process. Asymmetric extending vibrations of the carboxylate ion shifted to the higher wave numbers because, during the crosslinking process, the calcium ions substituted the Na ions in the sodium alginate. This shift was expected, as the atomic weight, density, charge, and radius of the cation were changed. The same behavior was recorded for PVA, the distinct peaks at 1648 and 1423 cm^−1^ shifted to 1660 and 1461 cm^−1^ ^65–67,69,70^. The cross-linking process between the hydroxyl (-OH) groups of sodium alginate (SA) and Ca^2^⁺ ions lead to distinct changes in the FTIR spectra, for the carboxylate (COO⁻) stretching bands. In the uncross-linked SA, the COO⁻ stretching bands typically appear in the range of 1650–1400 cm⁻^1^ (for asymmetric stretching and ν_asym COO⁻). After cross-linking with Ca^2^⁺ ions, these peaks shift to higher wavenumbers, indicating ionic interactions between carboxylate groups and calcium ions. Observably, for bioglass, the band present at 454 cm^−1^ is assigned to (Si–O–Si) bending vibration, which shifted to a higher wavelength and found at 465, 460, 458, and 456 cm^−1^ for bio-microspheres samples, respectively. This manner is mainly attributed to the addition of the drug, as the intensity of this peak increased as drug concentration increased ^4^. Likewise, the intensities of 1033 and 801 cm^−1^, which are assigned to the stretching mood of Si–O-Si, Si–O–P, and Si–O symmetric stretching of BIOGLASS, increased gradually as the drug content increased. Also, a higher crosslinking results in larger shifts in the bands (400–1650 cm^−1^) due to stronger ionic interactions.

**Fig. 5:**
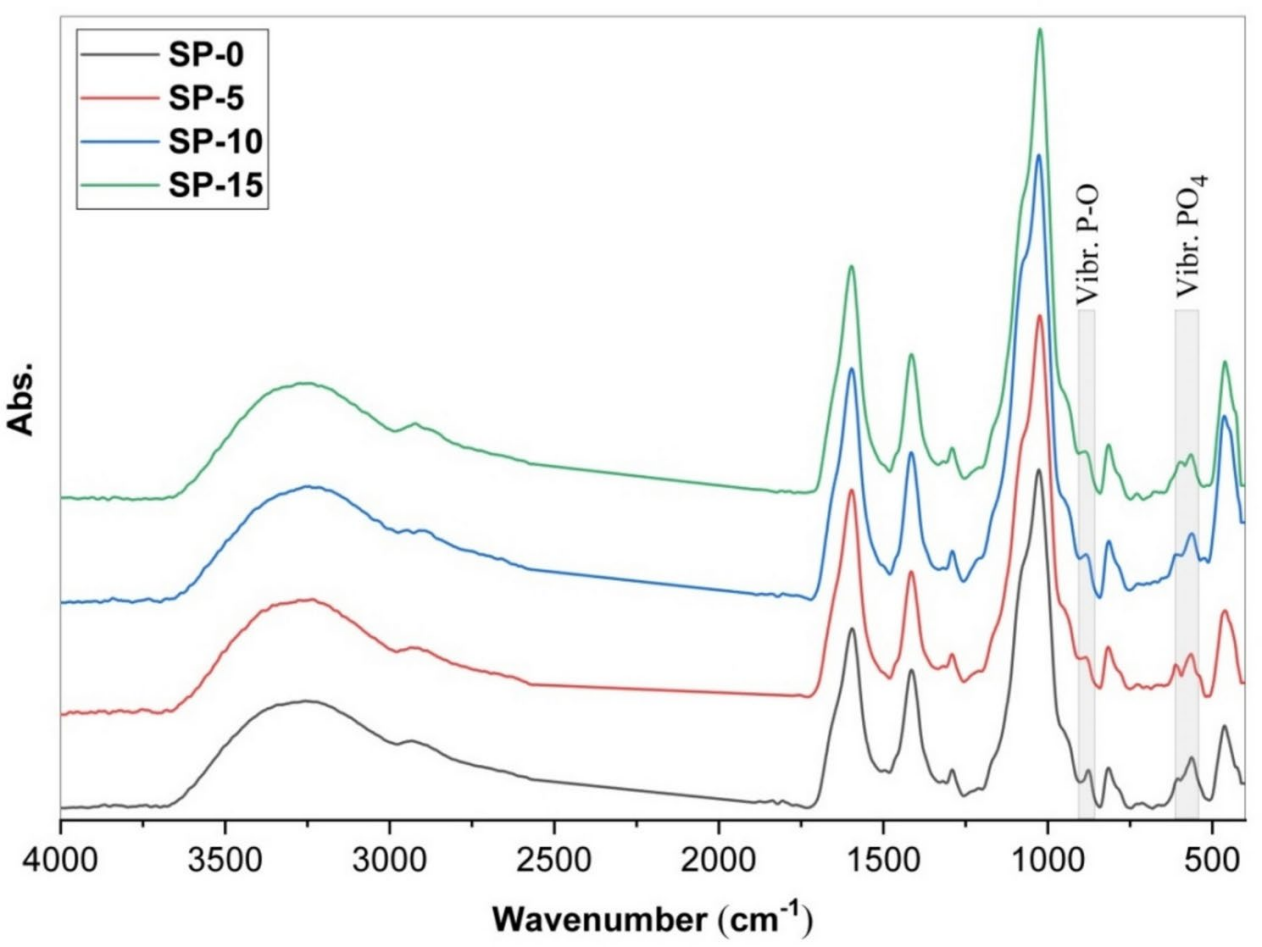
FTIR spectra of SP-0, SP-5, SP-10, & SP-15 microspheres nanocomposites after immersion in SBF (after 28 days).

Subsequently, the FTIR spectra of the bio-microsphere samples, soaking for 28 days in SBF solution, are presented in Fig. 5. In more particular reveal, The FTIR profiles of the fabricated microspheres display two new phosphate bands found at (565, 605) cm^−1^, and they are associated with the prolong bands of the ${\text{PO}}_{4}^{-3}$ a group within the crystalline segments of the phosphorus and the stretching groups of (P-O) appeared at 885 cm^−1^. These peaks confirm phosphate deposition and HAp formation. These results support the construction and growth of HAp ^4,50,65^. The change in FTIR broadening of the O–H stretching band (~ 3300 cm⁻^1^) is due to hydrogen bonding with Ca^2^⁺. These changes confirm the successful formation of Ca^2^⁺-alginate interactions, which contribute to the mechanical stability and bioactivity of the microspheres.

### Dielectric properties

The dielectric constant of materials measures the ability of materials to store the electrical energy in an electric field. In biological materials, this property is crucial because it influences how the material interacts with electromagnetic fields, which can have various biological effects. Biological materials contain both bound and free charges. As the electric field is introduced, the charges move and shift, resulting in conduction and polarization currents. However, Dielectric Spectroscopy investigates these effects by linking the dielectric features to the microscopic processes of polarization and conduction within the material ^76,77^.

**Fig. 6:**
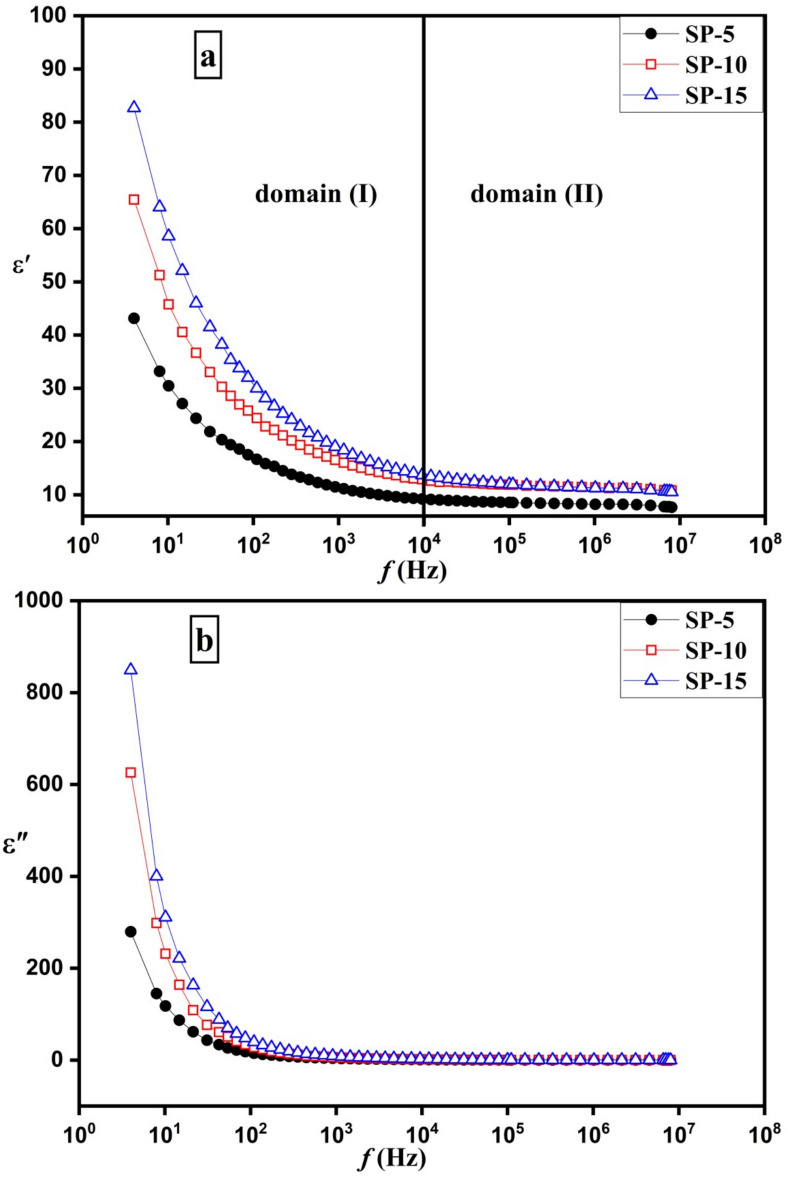
(**a**) The dielectric constant (εʹ)—frequency curve and (**b)** Dielectric loss (εʺ)—frequency curve of bio-microspheres nanocomposites.

Figure 6a illustrates the behavior of the dielectric constant of (SP-5), (SP-10), and (SP-15) in response to the external electric field frequency. As shown in Fig. 6a, below 10 kHz (domain (I)), the dielectric constant of all samples increases as the frequency decreases. This trend is indicative of dielectric relaxation in the samples concerning frequency^78,79^. In the low-frequency domain (I), the higher dielectric constant arises from various mechanisms of polarization, containing electrode polarization, interfacial polarization, and orientational polarization. Electrode polarization occurs because of the accumulation of free charge movers on the electrode surface, forming a capacitor layer, while the collection of charge carriers at the interlayer boundaries within the inhomogeneous components of the samples builds up a capacitor layer, resulting in interfacial polarization, Maxwell–Wagner (MW) effect ^80,81^. The orientational polarization results from the realignment of the internal dipoles with external frequency. At the high frequency domain (II) above 10 kHz, the dielectric constant has a neglecting change with increasing frequency, which can be attributed to the absence of the impact of the electrode polarization, interfacial polarization, and orientational polarization because they can’t follow the speed fluctuation of the frequency of the external field ^80–82^. The dielectric data follows a frequency-dependent trend that suggests deviations from ideal Debye behavior.

The current samples are composed of SA, PVP, BIOGLASS, and Amoxicillin/Clavulanic Acid acid-loaded with 5, 10, and 15%, which are electrically inhomogeneous components and therefore increase the total number of the interfacial dipole in the blended composites and hence increases the total dielectric constant of the samples, Fig. 6a. Growing the drug ratios in the blended nanocomposites raise the whole interfacial polarization, hence increasing the whole dielectric constant. Increasing the dielectric constant of materials indicates increasing the polarity of the blended composites and therefore increasing the antimicrobial effect ^76,77,83^. The correlation between the dielectric constant and antimicrobial activity may arise due to increased charge polarization, and ionic conductivity can influence drug release and bacterial interaction. Where the higher dielectric constant enhances the ionic movement that facilitates the controlled release of Amoxicillin/Clavulanic Acid. Also, the composite materials exhibit enhanced interfacial dipole interactions, which may affect bacterial adhesion and membrane integrity. Therefore, higher dielectric properties indicate better interaction between the polymeric matrix and bioglass, ensuring a more effective and sustained drug release ^76,77,83^.

The performance of the dielectric loss (εʺ) with frequencies of (SP-5), (SP-10), and (SP-15) (Fig. 6b) shows the same behavior as the dielectric constant, where it has a high value at low frequency owing to the effect of the different polarization such as electrode, interfacial, and orientational polarization besides the DC conductivity contribution to the dielectric loss. At high frequencies, the effects of such polarizations disappear, leading to a decrease in the dielectric loss ^84–86^.

**Fig. 7:**
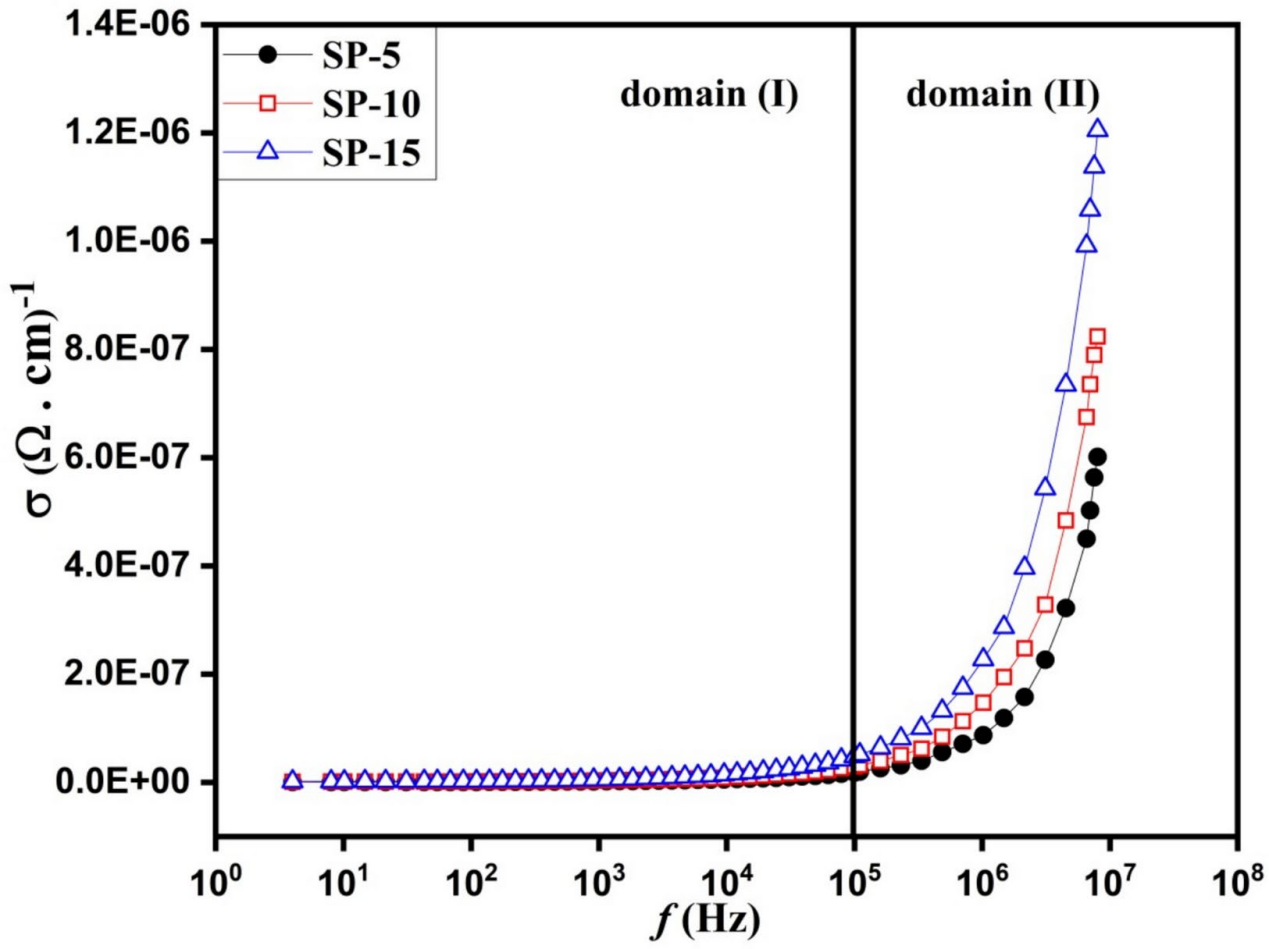
The AC conductivity of the microspheres composite (SP-5, SP-10, & SP-15) against the frequency of the applied external electric field.

Figure 7 presents the ac conductivity of the bio-microspheres nanocomposites as a function of frequency, while Table 1 presents the ac conductivity at selected frequency. Figure 7 displays the presence of two domains regarding the applied frequency. According to Fig. 7 and Table 2 the first domain (I) reveals DC conductivity, where the conductivity is almost stable with increasing frequency up to 100 kHz. At a frequency less than 100 kHz, the charge carriers transfer between the different sites through long-range hopping, where the DC conductivity has the major effect at this frequency range. At the higher domain (II) frequency, the conductivity of the composites shows an increasing behavior with increasing frequency. At domain (II) > 100 kHz, the conductivity is carried out through the localized motion of the charge carriers ^87–89^.

**Table 2:** The AC conductivity at specific frequencies.

Frequency range	Conductivity (σ) (S/cm)		
	SP-5	SP-10	SP-15
1 kHz	2.18832E-9	3.56273E-9	5.56659E-9
10 kHz	5.63274E-9	8.88532E-9	1.62703E-8
100 kHz	1.83378E-8	2.96109E-8	4.8329E-8
8 MHz	6.01496E-7	8.24117E-7	1.20494E-6

The conductivity behavior in both domains is closely linked to the material’s composition and microstructure. In domain I (low frequency), interfacial polarization effects dominate due to charge accumulation at phase boundaries within the bioglass-polymer-drug matrix, resulting in a relatively stable DC conductivity, where the bioglass provides the composites via mobile Ca^2^⁺ and Na⁺ ions, which trapped at the interfaces resulting in interfacial polarization that limiting the charge carrier movement and decreasing the total conductivity. In domain II (high frequency), conductivity increases due to hopping conduction, facilitated by the nanoscale distribution of bioglass particles within the SA-PVP matrix.

The Dielectric properties play a crucial role in cellular interactions, ion transport, and bioelectrical signaling, all of which are essential for osteogenesis and bone regeneration. Materials with a high dielectric constant can enhance charge storage and polarization, which may facilitate cell adhesion, proliferation, and differentiation by mimicking the natural bioelectric environment of bone tissue ^76,77^. Additionally, the observed frequency-dependent dielectric behavior suggests the presence of interfacial polarization, which can influence protein adsorption and cellular interactions, further supporting bioactivity. The AC conductivity of the microspheres also provides insight into their ionic mobility, which is essential for ion exchange processes involved in hydroxyapatite formation. By tuning these dielectric properties, the material may better support electrically responsive cell functions, making it more effective for bone repair applications.

### Antimicrobial activity

**Fig. 8:**
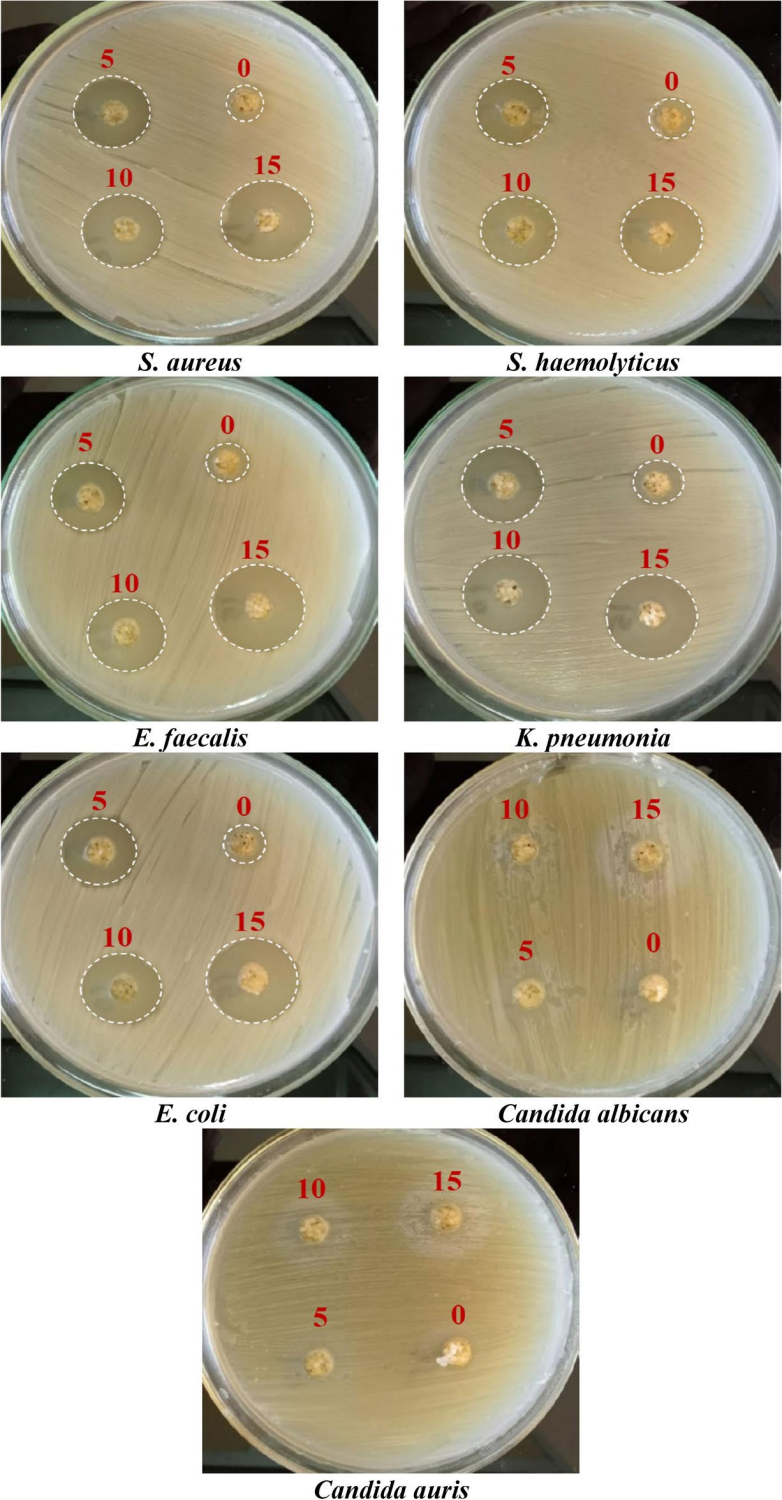
Antimicrobial activity of the prepared microspheres and their inhibition zones.

**Fig. 9:**
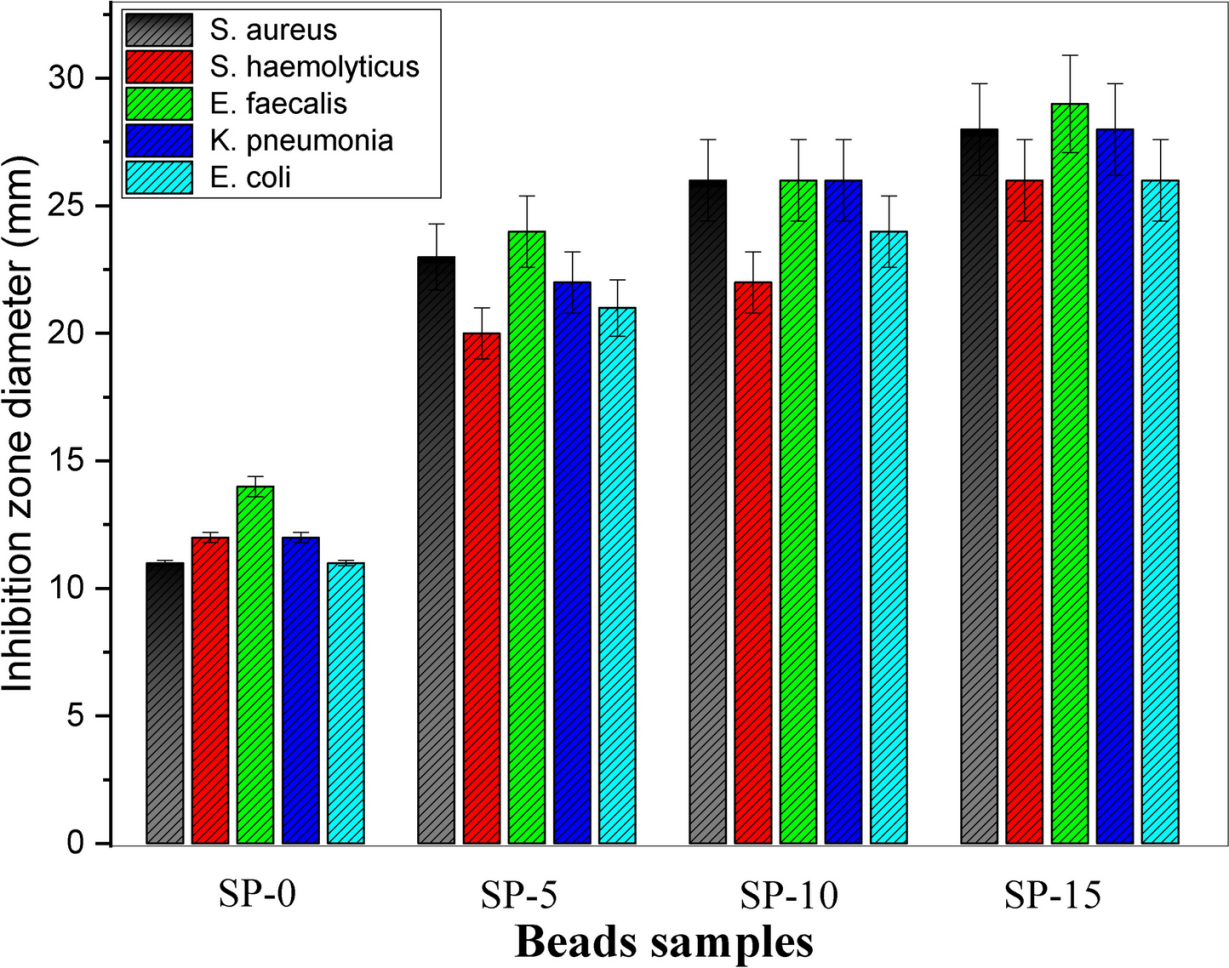
Antibacterial efficacy of the fabricated microsphere and their corresponding inhibition zones (Sample diameter 10 mm).

Figure 8 displays the antimicrobial evaluation of microsphere nanocomposites. The bacterial models were cultured at 32 ± 2 °C in a microbial incubator for 24 h, with shaking at 200 rpm, under sterile conditions. The clear inhibition zoon in Fig. 8 is induced due to the discharge of Amoxicillin/Clavulanic Acid from bioglass/SA + PVP nanocomposite microspheres. The antimicrobial activity of the nanocomposite microspheres is carried out for three times and the estimated inhibition zones are noted in Table 3 and displayed in Fig. 9. The inhibition zone diameters around the tried samples (SP, SP-5, SP-10, & SP-15) are as follows: (*Staphylococcus aureus*:- 11, 23, 26, and 28 mm), (*S. haemolyticus*:- 12, 20, 22, and 26 mm), (*Enterococcus faecalis*:- 14, 24, 26 & 29 mm), (*klebsiella pneumonia*:- 12, 22, 26, and 28 mm), and (*Escherichia coli*:- 11, 21, 24 and 26 mm), respectively. The data presented in Table 3 and Fig. 8 proves that bioglass/SA + PVP (SP-O) has an antimicrobial effect. The antimicrobial effect of the bioglass/SA + PVP (SP-O) may be raised from the Bioglass, where it may release Ca^2^⁺ and Na⁺ ions, which can alter bacterial membrane stability and promote an antimicrobial environment. The vibrant inhibition zone appearance around the prepared samples proved that the fabricated structure of Amoxicillin/Clavulanic Acid acid-loaded bioglass/SA + PVP (bio-microspheres) nanocomposites as a drug delivery carrier permits the antibiotic to discharge freely, and accordingly, the occurrence of the inhibition of the pathogenic microbes. It was observed that the performance of bio-microsphere nanocomposites was consistent concerning their influence on both + ve and −ve bacteria. Furthermore, the growth in antibiotic concentration resulted in the expansion of the clearing inhibition zone for both + ve and −ve bacteria. Also, increasing the antimicrobial effect can be attributed to the increasing polarity of the fabricated blended nanocomposite as predicated from increasing the dielectric constant ^76,77,83^. Additionally, the antimicrobial activity of the bio-microspheres is influenced by the surface area and functionality of both bioglass and SA-PVP. The high surface area of bioglass enhances ion exchange, increasing Ca^2^⁺ and Na⁺ release, which disrupts bacterial membranes. Additionally, the SA-PVP matrix modulates drug release and can influence bacterial adhesion ^90–92^. However, the clearing inhibition zone was largely absent in the fungal species, potentially representing a resistance response mechanism of the microorganisms against the used antibiotic. It may be because the Amoxicillin/Clavulanic Acid ratios present in the SA-PVP bio-microspheres nanocomposites are not suitable to hinder the propagation of the examined fungal or the microspheres form with SA-PVP polymers shielded the bioglass and drug ^2,92^. Moreover, it’s conceivable that bioglass and drugs are making bonds with the sodium alginate and PVP chains, hindering their release in adequate quantities^2,93^. The combination of bioglass, sodium alginate-PVP, and Amoxicillin/Clavulanic Acid improves bioactivity and antimicrobial efficiency, providing dual functionality for bone repair and infection prevention.

**Table 3:** Microbial sensitivity against pathogenic tested microorganisms.

Samples	Inhibition zone diameter (mm)						
	*S. aureus (Gram*^+^*)*	*S. haemolyticus (Gram*^+^*)*	*E. faecalis (Gram*^+^*)*	*K. pneumonia (Gram*^*-*^*)*	*E. coli (Gram*^*-*^*)*	*C. albicans*	*C. auris*
SP-O	11 ± 0.1	12 ± 0.2	14 ± 0.4	12 ± 0.2	11 ± 0.1	0	0
SP-5	23 ± 1.3	20 ± 1	24 ± 1.4	22 ± 1.2	21 ± 1.1	0	0
SP-10	26 ± 1.6	22 ± 1.2	26 ± 1.6	26 ± 1.6	24 ± 1.4	0	0
SP-15	28 ± 1.8	26 ± 1.6	29 ± 1.9	28 ± 1.8	26 ± 1.6	0	0

Sample diameter 10 mm.

## Conclusion

Development of advanced microsphere nanocomposites, including bioglass/SA-PVP incorporated with Amoxicillin/Clavulanic Acid, created using a sol–gel technique incorporated with a dropwise procedure for drug delivery and bone tissue engineering. The XRD and FTIR spectra of nanocomposites loaded with drug nano-microspheres revealed noteworthy results. X-ray diffraction (XRD) study established the amorphous trend of the prepared microspheres. SEM–EDX for the bio-microspheres reveals a well-integrated nanocomposite structure that confirms the sustained release of Amoxicillin/Clavulanic Acid, supporting ongoing protection against infection. Furthermore, FTIR investigation indicated that the existence of bioglass, oxide resulting in the breakdown of non-bridging Si–O bonds, transforming them into bridging Si–O–P and Si–O–Ca bonds within the sodium silicate configuration. The introduction of Amoxicillin/Clavulanic Acid supported the disruption in the Si–O–P–M bonds (where M expresses Si, P, and –OH), resulting in the production of (Si–O–Ca) & (P–O–Si–O–Ca) bonds within the bioglass/SA-PVP network structure. In addition, the SBF process produces additional P–O–P and P–O–Ca bridging. The comprehensive examination of the dielectric properties of the bioglass/SA-PVP loaded with the drug provides valuable insights into the complex dielectric properties of the investigated composites. The interfacial polarization has a great impact on the dielectric features, especially at the low frequency. The antimicrobial properties of microspheres were enhanced by adding higher contents of Amoxicillin/Clavulanic Acid. Also, the construction of an appetite layer via SBF condition was achieved. Generally, loading the Amoxicillin/Clavulanic Acid to the microsphere’s nanocomposites increased the bioactivity, and the antimicrobial efficacy with enhancing the SBF behavior provides higher bone tissue efficiency, reflecting its promising bioavailability. The combination of bioglass, PVP, sodium alginate microspheres and loaded with Amoxicillin/Clavulanic Acid results in advanced bioactive structure, antimicrobial, and drug-releasing properties, making Amoxicillin/Clavulanic Acid Bioglass/Sodium Alginate-PVP bio-microspheres suitable for bone engineering applications.

## Data Availability

The authors confirm that the data supporting the findings of this study are available within the article.
